# Machine Learning and 3D Reconstruction of Materials Surface for Nondestructive Inspection

**DOI:** 10.3390/s22166201

**Published:** 2022-08-18

**Authors:** Oleg O. Kartashov, Andrey V. Chernov, Alexander A. Alexandrov, Dmitry S. Polyanichenko, Vladislav S. Ierusalimov, Semyon A. Petrov, Maria A. Butakova

**Affiliations:** The Smart Materials Research Institute, Southern Federal University, 178/24 Sladkova, 344090 Rostov-on-Don, Russia

**Keywords:** computer vision, machine learning, nondestructive testing, technologies and systems, material surface reconstruction

## Abstract

During the steel pipeline installation, special attention is paid to the butt weld control performed by fusion welding. The operation of the currently popular automated X-ray and ultrasonic testing complexes is associated with high resource and monetary costs. In this regard, this work is devoted to the development of alternative and cost-effective means of preliminary quality control of the work performed based on the visual testing method. To achieve this goal, a hardware platform based on a single board Raspberry Pi4 minicomputer and a set of available modules and expansion cards is proposed, and software whose main functionality is implemented based on the systemic application of computer vision algorithms and machine learning methods. The YOLOv5 object detection algorithm and the random forest machine learning model were used as a defect detection and classification system. The mean average precision (mAP) of the trained YOLOv5 algorithm based on extracted weld contours is 86.9%. A copy of YOLOv5 trained on the images of control objects showed a mAP result of 96.8%. Random forest identifying of the defect precursor based on the point clouds of the weld surface achieved a mAP of 87.5%.

## 1. Introduction

Pipelines are essential strategic transport arteries for all countries around the world and play an important socioeconomic role for large energy and chemical companies. Within the framework of small subjects of states, pipelines serve as an integral tool for delivering critical natural resources and coolant to the final consumer: citizens. This type of transport has practical advantages, including environmental friendliness, the ability to operate under adverse weather conditions, the continuity of the resource delivery process, and optimization of the workload of the remaining modes of transport [1]. Pipelines are widely used today for the transmission and distribution of the most popular energy resources, such as gas and oil products, and for modern, promising, and more environmentally friendly initiatives, such as coal water slurry [1,2,3]. Restrictions imposed on the use and development of transport pipeline networks in some countries can significantly reduce general trends in developing the country’s technological and socio-economic potential [4]. Important aspects of the construction of such networks are the planning of their placement, the settlement of legal aspects of the construction of engineering structures, the quality control of the pipeline installation, and the compliance with all related bylaws and standards [1,5].

The primary materials for the installation of pipeline lines are steel, plastic, and propylene. Although each option has its advantages and disadvantages [6,7], the majority of popular choices for most of the networks currently under construction are steel. One of the most vulnerable places to install steel pipelines is the joint surface, which is made using welding technologies. Nondestructive testing methods (NDT) [8] were recently widely used to perform visual quality control checks of the surface of welded seams. The main nondestructive testing methods of the pipeline welded surface according to Table 341.3.2 of ASME B31.3-2004 [9] are visual inspection, liquid penetration inspection, magnetic particle testing, radiographic inspection, ultrasound testing, and eddy current testing. According to the data presented, the most effective method to recognize most weld imperfections is the radiographic method; however, visual evaluation allows you to identify approximately 70% of the types of defects. Based on the international standards ISO 5817:2014, ISO 6520-2:2013, and ISO/FDIS 9712-1999 in fusion welding parts, we can extract the key features of steel pipeline weld surface inspection and highlight the required list of defect detection accuracy.

There are many software and hardware systems and technologies for implementing methods for the nondestructive testing of girth welds in pipelines [10,11] that meet the requirements of the standards listed above. Radiographic and ultrasonic examination methods gained the greatest popularity [8,12]. However, both approaches are quite resource intensive. The high cost of equipment or consumables, significant time costs, and personnel qualifications are key factors that slow the pipeline construction process by contractors performing installation work. During pipeline line commissioning, licensed and accredited NDT laboratories are involved, which significantly guarantees the safety of the installations. In this case, the use of expensive equipment is fully justified. However, conventional contractors often require an affordable and sufficiently accurate means of assessing possible weld defects for a preliminary assessment of the quality of the steel pipeline being laid. Another important aspect of implementing control methods is their automation, which will significantly reduce resource costs during the construction process and ensure the optimization of the staff during line installation. Since the use of ready-made defect templates, industry standards for the enterprise do not provide high accuracy in defect assessment. The participation of an expert in the interpretation of sound spectra or X-ray images requires considerable time, considering the human factor, which does not always have a positive effect on the construction process: new methods are needed for decoding and classification of defects of the control object. Researchers are increasingly paying attention to the use of artificial intelligence technologies, which, together with sources of sensory perception of information about the progress of the welding process, allow the implementation of highly efficient algorithms for determining defects in the test object. First, we will highlight several stages to conduct circumferential weld inspections. A preliminary assessment is carried out directly during the construction process to obtain the minimum necessary to confirm information for the continuation of work. The next stage is the primary check, carried out before commissioning of the pipeline transport network. The final stage is a set of measures for the maintenance and inspection of pipelines in operation, among which are the methods of in-line inspection, such as magnetic flux leakage control (MFL), ultrasonic testing (UT), electromagnetic acoustic technology (EMAT), and eddy current testing (EC) [13].

In the further article text, an existing solutions mini-review will be presented, materials and methods will be identified, data preparation issues will be considered, the study main results will be analyzed, discussion will be held, and a conclusion will be made.

## 2. Existing Solutions Mini-Review

In this paper, we focus on the preliminary assessment stage of the visual control of the surface of steel pipeline girth welds. Thus, we considered several studies that allow us to determine the current state of the problem with respect to the implementation of determining defects regarding the most popular methods of NDT. For example, the paper [14], due to a detailed review of existing technological solutions, demonstrates the applicability of individual artificial intelligence methods and their advantages and disadvantages in organizing a defect classification system during the ultrasonic and radiographic studies of test objects. There are approaches to the analysis of radiographic images in which the authors proposed a hybrid approach that combines deep learning (DL) models based on the use of the AlexNet network and autoencoders. For example, these are artificial immune systems (AIS) [15]. According to the information provided by the authors, this approach makes it possible to significantly improve the accuracy of determining weld defects relative to the separate use of the indicated methods. The study [16] involves the initial extraction of the weld area from the radiographic image, which is analyzed using the traditional approach of extracting signs of a defect, such as threshold segmentation. In the future, it is planned to use the support vector machine (SVM) to detect blocks with defects. As a result, the authors determined the high classification ability of SVM, but noted the sensitivity of the results of applying this method relative to the original training datasets. Some research is focused on a more comprehensive approach to solving the problem, so in [17], a detailed description of the method is given to study weld defects using layered ultrasound arrays. The authors fully worked out the theoretical and practical aspects of the application of this method, including industrial use. The prospects for the use of automated ultrasonic testing (AUT) are outlined, and the results of multivariate analysis using principal component analysis (PCA) are presented. Some researchers are already concentrating their efforts on the development of existing methods and results. Thus, in [18], the authors define the procedure for X-ray image verification by qualified specialists and the need to automate this procedure. The study above used DL and transfer learning (TL) technologies based on pretrained deep convolution neural network models and a comparison of deep convolutional activation functions (DCFA). There are approaches to detect defects in steel pipeline welds based on the use of computer vision and machine learning algorithms. For example, in [19], the authors used the YOLOv5 algorithm and compared it with the two-stage algorithm to detect objects, Faster R-CNN. It should also be noted that the imaging setup in this study was a real-time X-ray imaging system consisting of a movement mechanism for the test tube, an X-ray machine HS-XY-225, a high-speed digital detector PS1313DX, and an image capture card [19]. In [20], attention was mainly paid to the detection of defects in the welds based on the localization of the defect by applying morphological filtering, further specifying the type of defect using a convolutional neural network (CNN) and the defect zone using SVM. Separate lines of research include the analysis of magnetic flux leakage imaging (MFL) images, where a gradient coincidence matrix (GGCM) was proposed as a defect recognition tool [21]. In some cases, to extract information on the presence of a defect from radiographic images, the Faster R-CNN algorithm is used, where the authors identified two extractors of internal features and evaluated their effectiveness in [22]. In general, the use of various types of images and spectra of sound sequences is one of the most sought-after areas of research in this area. Thus, the paper [23] reviews possible approaches to the analysis of X-ray and infra-red images of test objects and methods to determine defects based on the use of acoustic emission sensors and ultrasound. Although the study [24] is more focused on monitoring and analyzing the quality of the welds during pipeline operation, it allows you to get a description of such inspection methods, as the use of a three-axis high-definition MFL detector, an in-line ultrasonic crack detector, and the structure of an embedded detector with EMAT. In addition, we disclose the applications of technologies for electromagnetic eddy current testing and the verification of field measurements of alternating current (ACFM). The studies carried out in work [25] confirm the high efficiency of using the radiographic verification method under various conditions and allow the extraction of comprehensive information on welding equipment, the welding process, and the acceptable deviations in the evaluation of defects. Automatic systems that classify defects in steel pipe girth welds based on radiographic images using various machine learning models are the main proposal for the implementation of quality control verification in the installation work performed by welders. Thus, in [26], it is proposed to use the KNN and SVM methods to determine linear defects in pipeline welds. Such an integrated approach, according to the results obtained, guarantees an increase in the classification accuracy. In the study [27], the TL technology was implemented in conjunction with CNN. Here, traditional image processing techniques (IPT) were used to preprocess radiographic images. Descriptors extracted from the GDXray database using VGG16 and ResNet50 were used as data in the training sets of classifiers. The authors used random forest, support vector machine, and logistic regression as classification models. Regarding the results obtained, the authors present data on the increase in productivity and the high accuracy of these methods. Some studies offer alternative approaches to the calculation of weld defects, for example, the use of coarse set theory to create a classification system [28] or the implementation of linear control methods based on data management [13].

Based on the above information, we can conclude that the main methods of the nondestructive testing of girth welds of steel pipelines today are radiographic and ultrasonic testing methods. Furthermore, the wide possibilities of artificial intelligence methods offer an extensive toolkit for automating the identification and classification of possible defects in control objects. However, the purpose of this work is to create a simple and economical means of preliminary assessment of the quality of the installation work. In this study, a control system based on a visual inspection method is proposed, which in any case will not be able to recognize all types of weld imperfections. However, this tool will be able to detect in advance about 70% of violations of the weld manufacturing process, including cracks, lack of fusion, incomplete penetration, surface porosity or inclusion of exposed slag, surface finish, concave root surface, weld reinforcement, or internal protrusion, which in most cases guarantees increasing the efficiency and quality of work by contractors performing the installation of pipelines.

## 3. Materials and Methods

We chose a Raspberry Pi4 single-board computer as the main node to collect, broadcast, and preprocess visual information to develop the proposed software and hardware tool to inspect pipeline weld surfaces. This choice was based on the availability of the board, its low power consumption, extensive plug-in and expansion card capabilities, the presence of a built-in wireless module, and adequate performance for computing procedures and imaging research experience [29,30,31,32,33]. Since our project involves the connection of two camera modules to be able to build a depth map of the surface of the control object and an inertial measurement unit to determine the location and spatial orientation of the video information acquisition device, there was a problem with the presence of only one scalable coherent interconnect (SCI) port.

**Figure 1:**
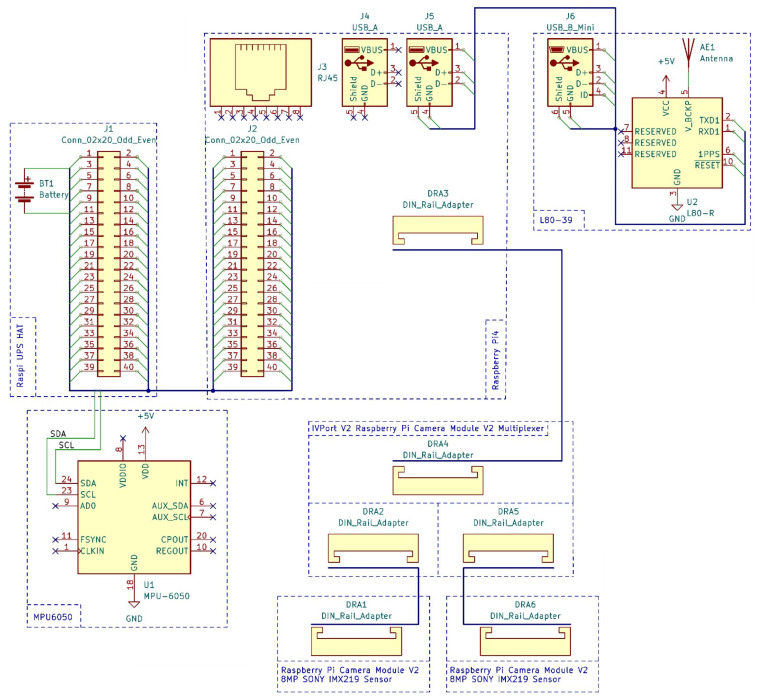
Connection diagram for Raspberry Pi4 modules and expansion boards.

This problem is proposed to be solved using a perfectly suitable IVPort V2 Raspberry Pi Camera Module V2 multiplexer [34]. This option allows you to connect to four camera modules simultaneously, which is essentially a hub from one SCI port to four. The main disadvantage of this board is the decrease in framerate for each of the connected cameras, since data capture is available from only one source connected through this board at a time. Since this board also has limitations on possible camera plug-ins, we chose the Raspberry Pi Camera Module V2 with 8MP SONY IMX219 sensor. The equivalent focal length of 33 mm, a sufficient lens aperture, and a wide range of supported video formats make it possible to use these modules in our project. The spatial orientation of the device is determined and assumed using the Kootek GY-521 MPU-6050 IMU modules for Raspberry Pi that operate on the IIC protocol with a built-in 16-bit AD converter. As a means of providing location data, the equipment is planned to use an L80-39 GPS module with a L80-39-based USB interface. The hardware platform is powered by another Raspi UPS HAT lithium battery expansion board, with 3.7 V specifications and a capacity of 2600 mAh. This reserve will allow the hardware of the proposed tool to operate for about two hours. The equipment connection diagram is shown in Figure 1. The circuit development was carried out with a cross-platform software package of the electronic design automation (EDA) class. It was also used to develop circuit diagrams and layouts of printed circuit boards.

Since it is supposed to study the girth welds on different diameters of pipes, the mechanical parts of the hardware platform suggest the possibility of adjusting the relative position of the cameras close to the object. The placement of onboard equipment requires the presence of a trolley with the possibility of uniform movement along the surface of the pipe. Transmission of preprocessed data to the user’s workstation is carried out by organizing the direct connection mode (ad hoc).

The methods implemented that are used in the development of the software part of the tool for diagnosing pipeline welds can be conditionally divided into several stages. The initial stage of the operation of the diagnostic tool is devoted to the collection of several data streams, including geolocation (data from the IMU and GPS module) and video data streams. Using the data received from the GPS module, a geotag is formed about the position of the pipeline joint diagnostics. This information will be used to generate a unique ID for each of the measurements. In addition, in the future, it will be possible to trace the control checks carried out on a digital map. Since, in our case, there are limitations to the possibility of synchronous video capture, the parameters of the initial video stream will be a resolution of 640 by 480 pixels at a frame rate of 15 frames per second. Since each module will perform the recording in turn, it is required to compare the frames of the video recording made by one module relative to another. When switching video recording channels, the delay is up to 1 s, which in some ways imposes restrictions on the speed of movement of the inspection tool trolley along the pipe surface. In our case, the IMU is needed to find the central shooting positions of the girth weld, which are sequentially different from each other by 45°, starting the report relative to the vertical axis of symmetry of the pipe. Time ranges are required to converge IMU readings with intermediate video recording measurement values, where the data stream from the measurement IMU is matched with the timeline of the measurement being performed, and then the required time values are extracted at the measurement control points. The time values obtained are then used to select and extract the appropriate keyframes from the video data streams, assigning a measurement position number. Direct extraction of frames from a video is performed and they are saved in an image file; then, a set of FFmpeg libraries from the Python procedural language is used. The effect of the impact of the encoder used in this set of libraries was determined in [35]. The name of the image file is derived from the number of camera modules, the measurement location, and the coordinates at a given time. Thus, in the output of this stage, we received two sets of image data, eight attachments each, where the file name contains additional information necessary for further use. The next step is to pre-process the image data files themselves. This section contains introductory material on the data preprocessing procedures. We will look at this in more detail in Data Preparation. In our case, the resulting visual data sets will be used in the future to solve two problems, the first is to identify and classify defects in the pipeline weld, the second is to make a three-dimensional reconstruction of the surface of the object under study with a color indication of areas with imperfections. Consequently, for each of the tasks to be solved, the preprocessing methods will differ. Since the measurement process does not take place under ideal conditions of illumination of the surface of the test object and there is a presence of extraneous noise in the image, it is proposed to use the median filter algorithm. The application of this approach can significantly improve the visual representation of the image while eliminating a significant amount of noise and equalizing the brightness of each image pixel [36].

**Figure 2:**
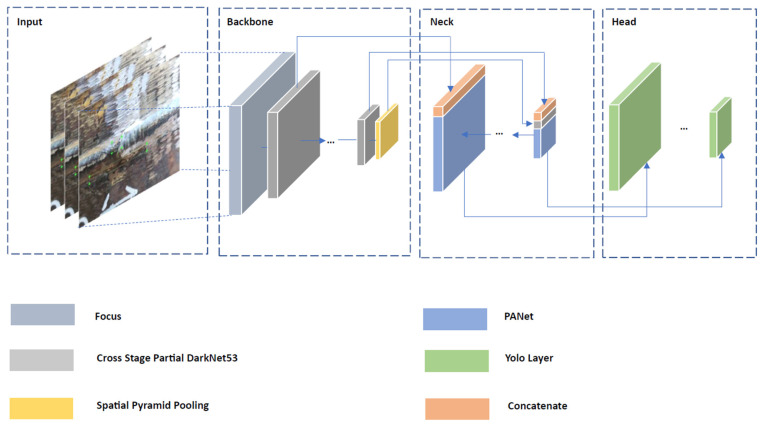
General network architecture of used YOLOv5.

The software implementation of the procedure for restoring the images obtained during the collection of information was performed using the OpenCV library in the Python programming language. Next, to implement the system used to classify weld defects, the resolution of the images in the studied sets was reduced. When designing a system to detect and classify defects in steel pipeline welds based on the nondestructive visual testing method, we noticed that most of the deviations depended primarily on the morphological properties of the object under study in the image. Therefore, an integrated approach to solving the problem was considered. Morphological characteristics were best demonstrated first by images of radiographic inspection of welded joints. In our case, the attribute space of such data sets is redundant, since, in addition to surface defects, they describe internal imperfections of welded joints. However, we discovered the possibility of extrapolating the results of the intellectual analysis of sets of radiographic images for the reliable identification of weld defects based on the visual method. Data sources for the study were the public radiographic image dataset GDXRay+ [37] and the radiographic and visual image datasets provided by LLC «Cyber Vision». YOLOv5 [38] was chosen as an algorithm to identify weld defects in images due to mosaic processing of graphic data, which allows qualitative extraction of features characterizing small rigid objects. The general network architecture used by YOLOv5 is shown in Figure 2.

**Figure 3:**
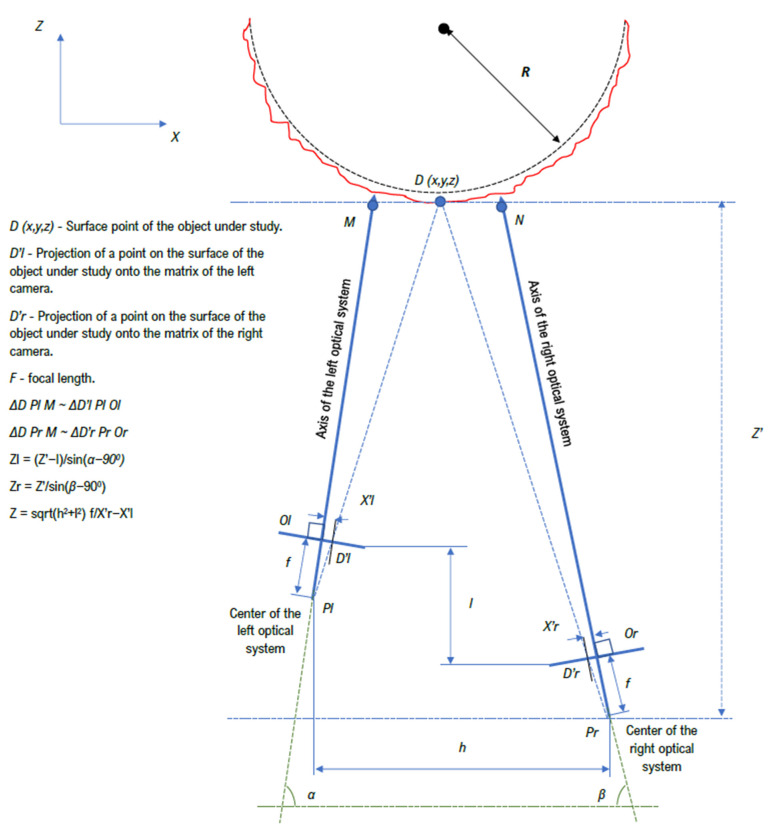
General view of the process of restoring the depth of an object based on a binocular approach.

The main large parts of YOLOv5 are backbone, neck, and head. The first one extracts and forms image features with different detail levels. The next one is used for feature aggregation. The last makes predictions based on features from the neck. For backbone used Cross Stage Partial DarkNet53 (CSP DarkNet) with spatial pyramid pooling. This serves to increase the receptive field at separating features without compromising performance. For the neck, the PANet solution is used, which provides an adaptive pool of features connecting the mesh with all levels of features, for uniform propagation in the following steps. The Yolo layer in the head implements dense prediction for a one-stage detector type. In general, the choice in favor of YOLOv5 was also made on a few considerations. Thanks to CSP DarkNet, this algorithm has high speed and accuracy of inference. PANet allows for more efficient low-level feature propagation, and the dense prediction layer makes it possible to implement a multiscale prediction, which is quite useful for the condition of various morphological characteristics of weld defects. In addition, in the study [19], the authors carry out comparative tests on a number of approaches to one-stage and two-stage detection, where YOLOv5 showed excellent results. It is especially worth noting the superiority over Faster R-CNN in terms of speed, accuracy, and final weight of the model. Based on the data used in [19], we considered it possible to extend their experience to our study. The ability to detect and classify in real time, the low weight of the trained model, the speed and issuing efficiency of the predictions, the simplicity of data labeling, and training make YOLOv5 an advantageous candidate for solving our problem. This algorithm was trained twice. In the first case, a Gaussian blur with a radius of 5 was applied to the sets of radiographic images, and the edges of the objects were extracted using the Prewitt operator. Additionally, we made changes to the data labeling by eliminating hidden defects that are inaccessible to the visual method of control. The resulting modified data sets were used to train YOLOv5, where the contours of the test objects extracted from conventional weld images using the same Prewitt operator were used as a test set. In the second case, data characterizing surface defects were selected from the sets of radiographic images and the model of the YOLOv5 algorithm was trained, which was further trained on the labeled data of ordinary images. This was done on the basis that there were about 8000 examples of radiographic images, divided into 6 classes of welding defects, and there were about 1500 ordinary images, which could not guarantee a confident prediction of the presence of a defect in itself. In the process of shooting controlled seams with two cameras, we restored the depth of objects; a general view of this procedure is shown in Figure 3.

To reconstruct the depth map, algorithms for searching for local features in images were used, since the algorithms for searching for global matches are more computationally expensive, and this contradicts the proposed concept of the approach. The depth maps obtained were converted to point clouds on the surface of the welded joints of steel pipelines. The extracted point cloud data were used to detect and classify weld defects at the level of their morphological characteristics. For this, a modified method was used with further analysis based on the random forest model [39,40,41]. We implemented and developed the procedure for the three-dimensional reconstruction of the surface of the pipeline weld.

Because of the shooting of images is performed in key positions, there are overlap zones (repeating visual information in two images) and distortions of the weld geometry relative to the shape of the object under study; when merging eight images of the weld, it is necessary to refine the overlap zones and correct the shape of the weld on the image. Standard tools provided by the OpenCV library were used to implement the image splicing procedure in software. After obtaining two spliced images relative to visual data sets from two multidirectional camera modules, it is required to build disparity and depth maps. Next, we use the software implementation of the computer vision algorithm, efficient large-scale stereo matching [42]. Based on the results of third-party research conducted in other areas [43], quite good results are guaranteed in our case, with relatively low complexity of implementation. After obtaining disparity and depth maps, it becomes possible to build an red, green, blue, depth matrix (RGBD) for the image of the weld. Then, using geometric arguments, we implement the process of converting the screen coordinate system to a Cartesian coordinate system. This idea was born from the reverse engineering results presented in [44]. However, despite obtaining a point cloud of the weld surface, it is worth recalling the geometry of the test object, the pipe, of which, as a result, the three-dimensional reconstruction should be a three-dimensional ring. We used the values of the radius of the curvature of the pipe relative to the size of its diameter as a correction factor that increases with each step for the location of each cloud point in the Cartesian coordinate system. Then, the reconstruction of the surface mesh [45], with respect to the coordinates of the point cloud, is assumed. The software implementation of this procedure was performed through a convolutional neural network with an integrated voxelization graph (LV-GCNN) [46], which allows reconstructing the surface without visible losses. The resulting digital model is then divided into sections according to the initial shooting positions.

## 4. Data Preparation

In this study, a total of four initial datasets were used. This is a public GDXRay data set, or rather, a part of it containing X-ray images of welded joints. A set of weld radiographic images (8000 images) and a data set of weld photographs (1500 images) were provided by Cyber Vision LLC. In addition, we collected a dataset of point clouds of steel pipeline weld surfaces (100 surfaces). In addition, on the basis of existing sets of graphical data, a set was created that contains images of the extracted contours of the welds. In the process, all datasets were labeled or relabeled and pre-processed.

**Figure 4:**
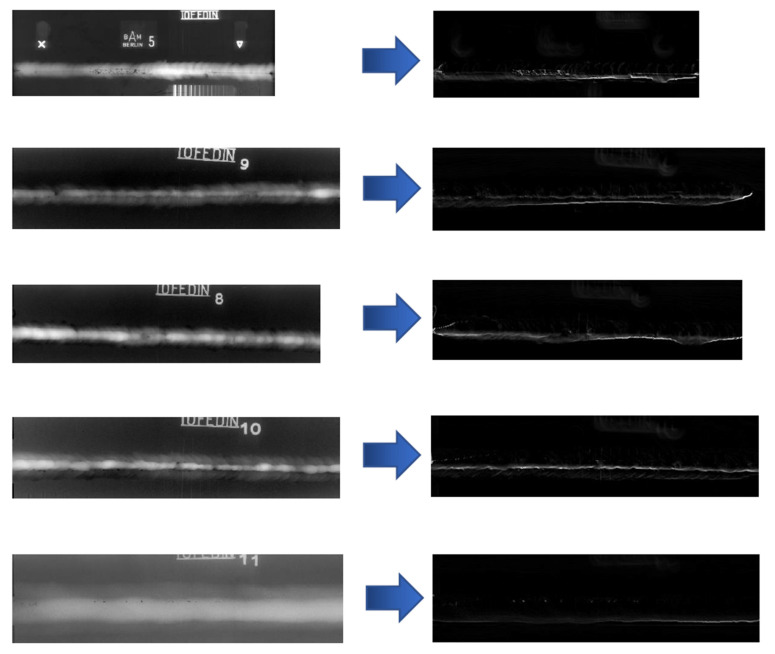
Results of preprocessing images of welded joints radiographic inspection.

We selected two data preprocessing scenarios based on the problems of detection and classification of defects and the three-dimensional reconstruction of the surface of the test object. As part of the first procedure implementation, images of two types were used: the usual visual and radiographic representations of the test object. The radiographic images were subjected to a Gaussian blur procedure with a 5 radius and further extraction of the edges of the objects in the image using the Prewitt operator. The results of this processing are shown in Figure 4.

For processing graphic materials of visual data sets of weld images, the methods used in our case can be divided into setting the basic resolution, filtering (noise removal and brightness equalization), segmentation, and color space transformation. A resolution of 224 × 224 pixels was defined as the reference resolution for sample images. For this, software procedures for cropping frames and reducing their resolution were implemented. At this point, image data pre-processing procedures were suspended in favor of markup. The marking of the images was completed manually by an expert experienced in the visual assessment of weld seams. The procedure for labeling images of welds was carried out using the LabelImg software, which has a clear graphical interface and wide functionality for marking data sets. In the process of marking these images of welded seams of steel pipelines, we were guided by two international standards, in particular ISO 5817-2021 and ISO 6520-2-2021.

**Figure 5:**
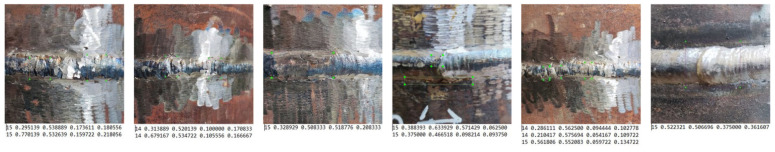
Samples of marked welds in steel pipelines images.

Based on the standards considered, six main types of welding defects and their descriptors were identified for the implementation of the visual method of nondestructive testing. The identified types of defects include: (1) cracks; (2) cavities; (3) solid inclusions; (4) non-fusion; (5) form defects; and (6) other defects. Examples of marking images of welded seams of steel pipelines are shown in Figure 5.

At this stage, the first set of visual data was formed, characterizing the defect area of the test object based on the color space of RGB images, which is useful for identifying such defects as, for example, inclusions.

**Figure 6:**
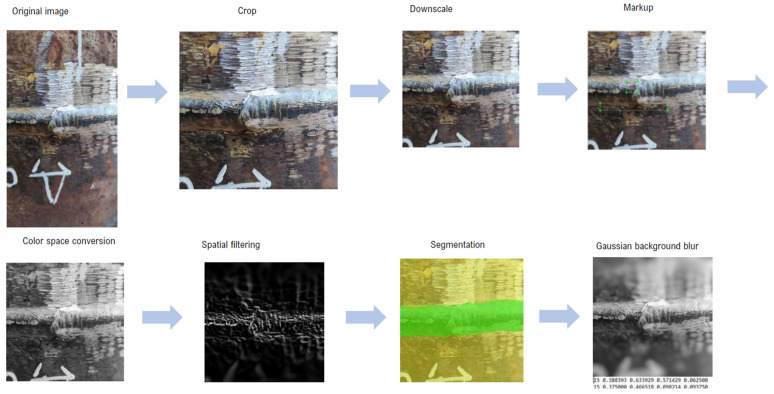
Stages of preliminary processing of image data.

For better detection of other types of defects, it is necessary to highlight the most informative features of visual data. For this, three stages of preprocessing graphic data are proposed. All procedures for pre-processing image data are shown in Figure 6.

**Figure 7:**
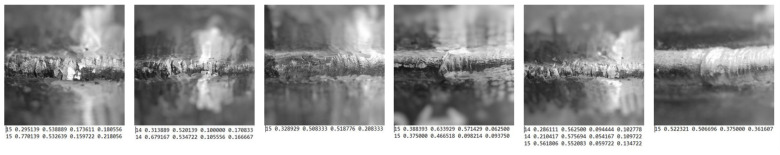
Examples of preprocessed and labeled image data.

In the first stage, the image color space was converted to grayscale. This procedure was implemented using the multiparadigm Python programming language and the scikit-image library. The next step was dedicated to segmenting the image to highlight the contours of the weld against the background of the steel pipe. For this, a spatial filtering method based on a sliding mask with a dimension of 3 × 3 pixels was applied, and a Gaussian blur with an intensity of 3 was previously used to eliminate excess noise. The coefficients of the mask square matrix were determined using the Prewitt operator. After successful selection of the weld contour, blurring of the image background was applied using the Gaussian function and an intensity of 10. Examples of the images obtained are shown in Figure 7.

The second scenario does not include reducing the resolution of the original image, but implements the procedure for merging all images within each of the two sets. The Random Sample Consensus (RANSAC) and Scale-Invariant Feature Transform (SIFT) algorithms were used for image splicing, which reveal local features with which you can check the similarity of the original images in the set. In addition, we used the SIFT algorithm to calculate and represent the three-dimensional surface points of the testing object based on two spliced images. The pseudocode for the software implementation of this function is given in Algorithm 1.
**Algorithm 1.** Calculation and presentation of points of a three-dimensional controlled object/procedure CalculatePointCloud(imgL, imgR).**INPUT**: Two images of calibrated cameras **OUTPUT**: Point cloud
        imgL:= ConvertColor(imgL, GRAY)
        imgR:= ConvertColor(imgR, GRAY)
        keypoints_1:= SiftDetectAndCompute(imgL)
        keypoints_2:= SiftDetectAndCompute(imgR)
        keypoints_1, keypoints_2:= Match(keypoints_1, keypoints_2)
        F:= FindFundamentalMat(keypoints_1, keypoints_2)        pointCloud:= Triangulation(F, keypoints_1, keypoints_2)
        **return** pointCloud
**end** procedure

Additionally, within the study framework, an approach was proposed and implemented to assess the defectiveness of the welds based on the analysis of a cloud of points on the surface of the test object using random forest. To do this, point cloud data was collected using the Intel Realsense D435i intelligent sensor camera, followed by the extraction of the required values from the bag file. It was these data that were used later to train the random forest. The real test data sample was formed by algorithmic processing of two video data streams and the subsequent transformation of the obtained depth maps into point clouds. A fragment of the structure of the received point cloud data is shown in Table 1, and the raw data and other open results of the ongoing research are available in the public repository of projects at the link: https://github.com/cybervllc/real_soft (accessed on 22 July 2022).

Marking of these point clouds was carried out by selecting a sequence of points belonging to the defective zone of the weld or defining other morphological characteristics that do not meet the requirements of the international standards in this area. As a result, each investigated weld fragment had 11,000 to 13,000 points, and the average number of points to describe the defective zone was in the range of 1500–2000 points from the entire fragment array.

## 5. Results

The proposed approach to assessing the defectiveness of a weld based on processed video data streams is to implement a deliberative procedure of three trained machine learning algorithms, including YOLOv5 to identify anomalous contours of objects, YOLOv5 to detect defective zones in images of a weld, and random forest to analyze spatial data point clouds of the surface of the welded joints of steel pipelines. The deliberative procedure basis is the principle of two of three. This means that at least two of the three algorithms must detect a defect in the object under study, even if they identify a different type of deviation. The training of all test models took place on a stand that included a discrete adapter Nvidia Quadro RTX8000 with a memory capacity of 48 GB, an Intel Xeon E5-4655 v3, and 128 GB RAM with the Linux Ubuntu 18.04.6 operating system on board. To train the YOLOv5 first case, the hyperparameters set given in Table 2 was used.

**Figure 8:**
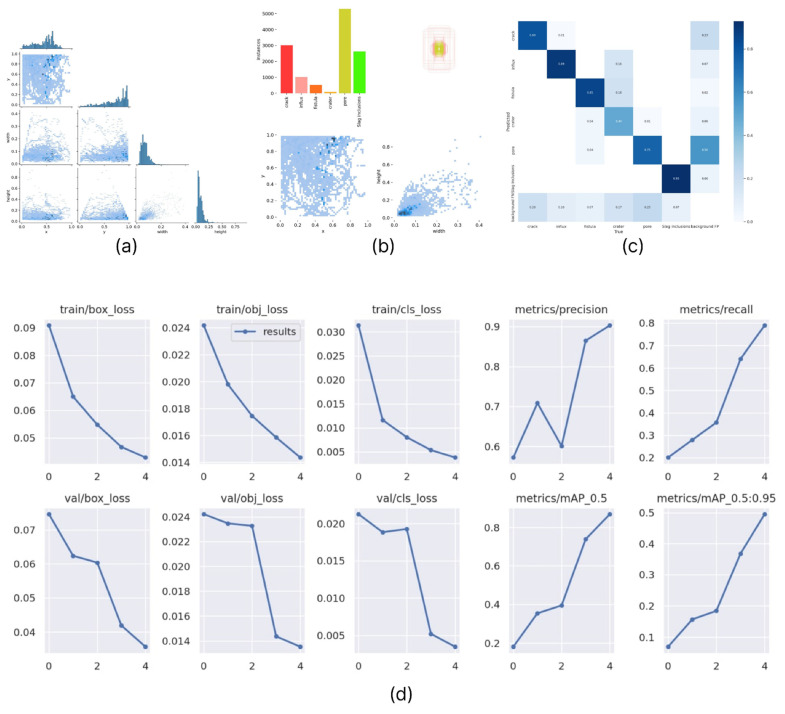
First case model training and validation metrics for a set of counter images.

The results obtained during the first computer vision algorithm training to detect defects based on the image contours of a steel pipeline weld are presented in the graphic materials below in Figure 8. Here, Figure 8a shows a correlogram of ROI labels, which is a group of two-dimensional histograms showing pairwise projections of the data axes according to the “each with each” principle. Figure 8b contains information about the distribution density of image labeling parameters relative to all available classes. The confusion matrix of first case YOLOv5 is given on Figure 8c. The upper row Figure 8d shows the model training metrics. Specifically, in order, root mean square error (RMSE), binary cross-entropy, entropy, precision, and recall. The bottom row Figure 8d shows the same set, only for model validation.

**Figure 9:**
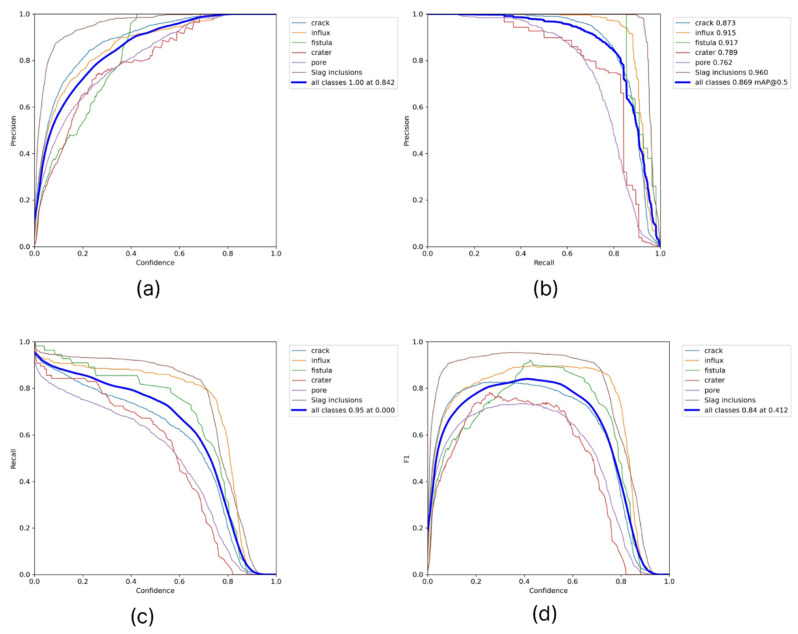
Mutual dependencies of the model metric indicators used (**a**) precision–confidence, (**b**) precision–recall, (**c**) recall–confidence, and (**d**) F1–confidence.

Additionally, for a qualitative result obtained from the examination, we collected pairwise graphs of the dependence of the quality metrics of the algorithm used, such as precision, recall, f1, and confidence (Figure 9).

This result allows us to assert the high accuracy and stability of the work of this algorithm. The training of the second copy of YOLOv5 took place in two stages. At the first stage, the algorithm was pretrained on radiographic control images. At the second stage, it was further trained on preprocessed and marked images of welded steel pipes. To train the YOLOv5 s case, the hyperparameter set given in Table 3 was used.

**Figure 10:**
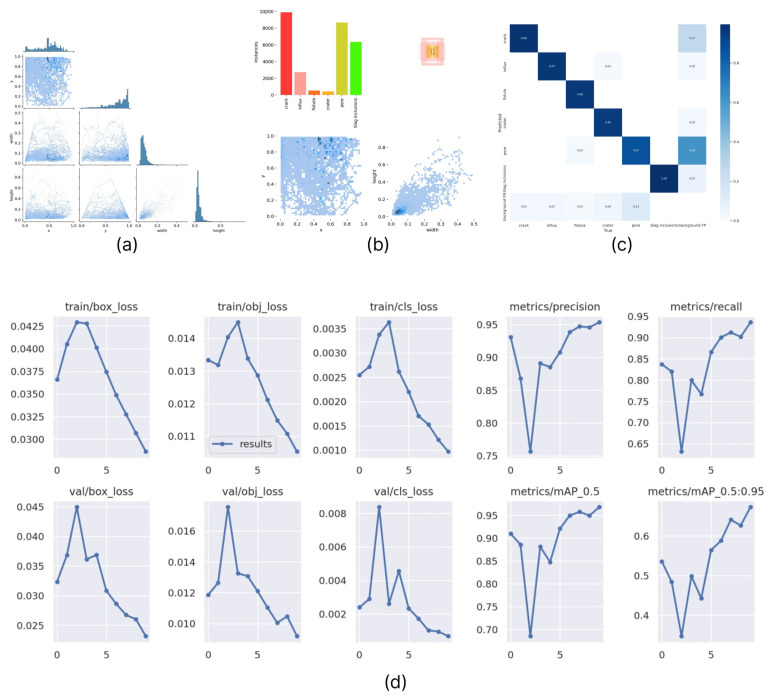
Second case model training and validation metrics for a set of counter images. (**a**) Correlogram of ROI labels; (**b**) Distribution density of image labeling parameters; (**c**) Confusion matrix; (**d**) Standard set YOLOv5 training and validation metrics.

The final metrics to train the algorithm and the quality of the model are given below. The Figure 10 structure is identical to the Figure 8 structure, except that it contains an evaluation of the YOLOv5 second case.

**Figure 11:**
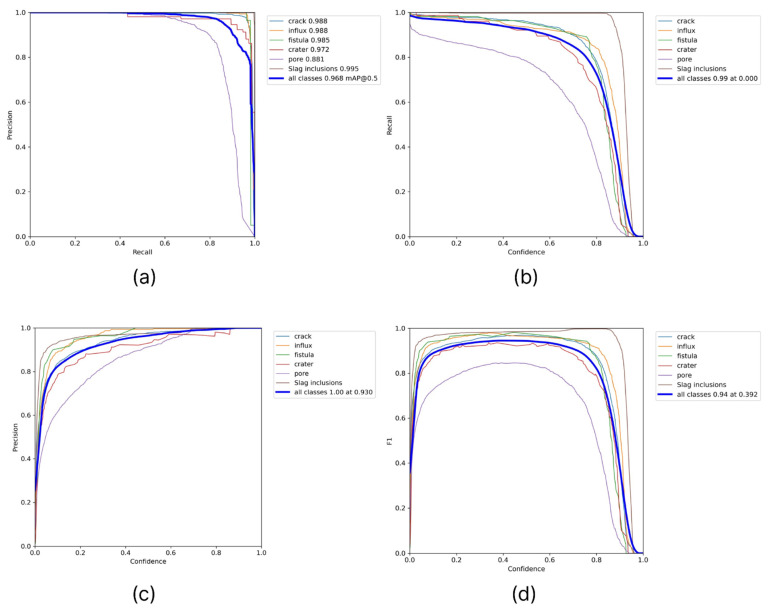
Mutual dependencies of the model metric indicators used in the second case: (**a**) precision–recall, (**b**) recall–confidence, (**c**) precision–confidence, and (**d**) F1–confidence.

In addition, as in the first case, graphs of mutual dependencies of the metric indicators of the model used for its characterization were prepared (Figure 11).

**Figure 12:**
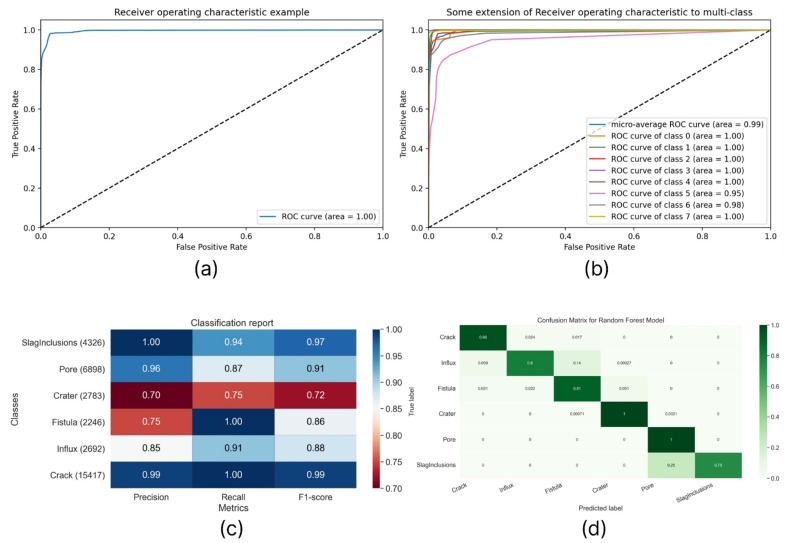
(**a**) Average ROC (**b**) ROC area to multilabel classification, (**c**) PRA map showing dependencies average precision, recall, and F1-score metrics. (**d**) Confusion matrix.

In addition to these algorithms for the detection and identification of defects in welded joints, an approach based on the analysis of the obtained point clouds of the object surface under study was used. Strictly speaking, we determined each point potential from the set belonging to a possible defect using the random forest algorithm. In this case, the point clouds belonging to surface defects and described above served as the initial data set. This approach concept is as follows: we consider independently each point from the points cloud corresponding to the one-object measurement and present the probability that this point belongs to one of the six defects. Next, we integrate the number of points that belong to the same defect in the areas of the surface under study, having previously clustered them. If the checksum of the number of points per potential defect exceeds the threshold value, the system determines this as a precursor of a weld defect. Since we know in advance the geometry of the control measurements and the survey scale, the values of the threshold values of the control sums were chosen on the basis of the average geometric dimensions of real defects and are unique for each type of defect. Data on the training and testing processes of the random forest algorithm are shown in Figure 12.

As a result, we prepared and trained three algorithms, YOLOv5 to detect defects based on images of weld contours with an mAP@0.5 value of 86.9%, YOLOv5 to detect defects in welded joints based on images of the test object with an mAP@0.5 value of 96, 8%, and random forest to detect the belonging of individual points from the cloud to defect areas with mAP@0.5 of 87.5%. These indicators cannot be directly interpreted to assess the quality of the defect detection and classification system. Therefore, in the discussion paragraph, we describe the full-scale experiment we conducted to assess the quality of the system operation.

## 6. Discussion

During the study and assembly of the prototype, we identified limitations in the use of the proposed software and hardware platform for the implementation of the visual inspection of girth welds in steel pipelines. Among them are the limitations determined by the very procedure for checking the quality of the work performed, which consist of the range of diameters of the investigated pipes and the need for a uniform movement of the trolley with on-board equipment along the surface of the object under study at a low speed. The main disadvantage of the proposed approach is an incomplete list of the types of defects identified. However, this method of nondestructive testing does not involve the determination of such defects as internal porosity, internal slag inclusion, tungsten inclusion, or elongated indication and undercutting. Determining these types of defects certainly plays an important role in the safer operation of the pipeline line; however, it requires the implementation of expensive and resource-intensive methods; therefore, in the context of this work, we present our result in the field of a preliminary assessment of installation work performed directly in the construction process. The hardware of the proposed monitoring tool is characterized by the low cost of the individual components, which guarantees their easy replacement in case of failure, and low power consumption, which guarantees low installation dimensions with sufficient battery life. The software implements a system to determine and classify defects, as well as generates final reports on the inspection carried out with a three-dimensional reconstruction of the weld surface under study. It is based on a set of methods and approaches tested by other researchers, with the introduction of the authors’ corrections regarding the problem being solved and the area of research under consideration. The use of computer vision algorithms, machine learning models, and neural networks makes it possible to organize a procedure for the automated detection of defects in the test object with sufficient sensitivity and accuracy, which contributes to the application of this tool in practice. Based on the results of this study, 100 full-scale experiments were carried out to control the quality of welded joints with the involvement of a human expert from a certified laboratory of nondestructive testing methods. All experiments were carried out in clear sunny weather during the first half of the day at one site of installation working on laying a gas pipeline. As a result, the expert identified two serious violations of installation technology, which cast doubt on the possibility of further operation of the line with the assignment of the required category of the facility, and about seven minor defects that allow further use of the pipeline. The system identified approximately ten cases of violations in the production of pipeline welds, two of which were classified as potentially dangerous by the expert, and four that were classified as minor. The expert marked the remaining four detections as false positives; in addition, he noted three not considered by the system. Unfortunately, at the time of writing this article, there were no other objects for nondestructive testing of butt joints of steel pipelines in the work of LLC «Cyber Vision», and we were unable to expand the statistical sample of the actual system performance.

## 7. Conclusions

In, the proposed study several results were obtained. We assembled a working prototype of a hardware platform based on Raspberry Pi modules and expansion boards. Several scenarios for data preprocessing and training sets formation were proposed and successfully implemented, allowing extrapolating, and at some stages, the available X-ray images of welds to detect and classify defects based on extracted frames from a conventional video sequence of the visual nondestructive testing method. To implement the system for detecting and classifying defects, the application of the system of computer vision algorithms and the machine learning model was chosen, which provides the prediction fusion at the deliberative procedure level. The trained models received the following indicators: The YOLOv5 first case trained on weld contour images extracted from radiographic and conventional images of the test object achieved a mAP@0.5 value of 86.9%. The second case YOLOv5, trained on radiographic inspection images with no markings on internal defects and retrained on conventional photographs of defective welds, received mAP@0.5 96.8%. Random forest, which determines the probability that a point belongs to a defective zone from the weld surface cloud points, reached mAP@0.5 87.5%. In addition, we conducted a full-scale experiment in the field, in which the proposed hardware and software platform showed its stability. We fully agree that our proposed approach is inferior to existing solutions based on radiographic and ultrasound examinations. However, the goal of our work was to create a convenient and fast method for a preliminary assessment of the quality of welding work “in the field.” In most cases, this will have a significant economic and temporary effect in the case of early detection of weld defects. In addition, we outlined a further trajectory of work in this direction. It is planned to consider the capabilities of Inceptionv4 in solving the identified problems, as well as to adjust the YOLOv5 structure by adding convolution from GoogLeNet/Inception to it in order to improve the accuracy and speed of defect detection. Additionally, among other things, the performance of the Raspberry Pi4 node in some tasks does not meet expectations, so we plan to select another hardware platform in accordance with our vision of an economical product.

## Figures and Tables

**Table 1:** Data structure of the point clouds of the weld surface of the steel pipeline.

	x	y	z	nx	ny	nz	Red	Green	Blue	Alpha
**0**	−0.27675	2.1516	3.5621	4.520603	4.28289	3.83560	20	47	103	255
**1**	−0.27750	2.1557	3.5664	4.749300	3.10760	3.18440	36	35	96	255
**2**	−0.28250	2.1602	3.5701	3.159200	2.98990	4.15230	45	51	110	255
**3**	−0.28900	2.1697	3.5709	4.062900	4.11780	2.99978	23	49	99	255
**4**	−0.28947	2.1697	3.5718	4.131200	4.56719	4.36810	19	37	106	255

**Table 2:** Values of YOLOv5 first case hyperparameters during training.

Parameter	Meaning
Number of epochs	50
Batch size	64
Learning rate	0.01
Momentum	0.937
Weight decay	0.005
Image caching	Yes

**Table 3:** Values of YOLOv5 s case hyperparameters during training.

Parameter	Meaning
Number of epochs	25
Batch size	64
Learning rate	0.01
Momentum	0.937
Weight decay	0.005
Image caching	Yes

## Data Availability

Not applicable.
